# Supplementary material for: Low-salinity medium for large-scale biomass production of the marine purple photosynthetic bacterium Rhodovulum sulfidophilum
Source: PLoS One. 2025 Jun 24;20(6):e0321821. doi: 10.1371/journal.pone.0321821 (PMC12186965; doi:10.1371/journal.pone.0321821)
Supplement: S1 Table — Data are presented for four independent 10 L batch cultures (n = 4). P values were obtained from one-way ANOVA (Dunnett’s test) (GraphPad Prism 9) by comparing marine broth (MB) with natural seawater (NSW) and artificial seawater (ASW) based media. (PDF) [file pone.0321821.s001.pdf]

S1 Table.

		MB	NSW + 0.1% YE + 0.5% Peptone	ASW + 0.1% YE + 0.5% Peptone
Fresh cell yield ($g\ L^{-1}$)	1	7.23	5.75	5.62
	2	7.82	4.80	5.59
	3	6.92	3.96	4.64
	4	5.99	4.10	4.26
	Mean	6.99	4.65	5.03
	SEM	0.38	1.10	0.34
	p		0.0033	0.0095
Dry cell yield ($g\ L^{-1}$)	1	1.03	1.17	0.93
	2	1.04	0.95	0.98
	3	0.95	1.01	0.88
	4	0.82	1.00	0.74
	Mean	0.96	1.03	0.88
	SEM	0.05	0.23	0.05
	p		0.4983	0.4646
