# Supplementary material for: Low-salinity medium for large-scale biomass production of the marine purple photosynthetic bacterium Rhodovulum sulfidophilum
Source: PLoS One. 2025 Jun 24;20(6):e0321821. doi: 10.1371/journal.pone.0321821 (PMC12186965; doi:10.1371/journal.pone.0321821)
Supplement: S2 Table — Data used for the growth curve (OD660) of R. sulfidophilum in ASW supplemented with 0.1% yeast extract and 0.5% peptone in decreasing concentrations of ASW, i.e., 100%, 90%, 80%, 70%, 60%, and 50% which correspond to 3%, 2.7%, 2.4%, 2.1%, 1.8%, and 1.5% salinities respectively (Fig 1a). Data are presented for three independent 15 mL batch cultures (n = 3). (PDF) [file pone.0321821.s002.pdf]

S2 Table.

		OD_{660}						
		0 hours	24 hours	48.5 hours	72 hours	96 hours	128 hours	176.5 hours
100% ASW	1	0.14	0.54	1.00	1.19	1.48	1.71	1.92
	2	0.11	0.52	1.08	1.18	1.50	1.79	1.74
	3	0.13	0.51	1.00	1.11	1.45	1.79	1.80
	Mean	0.13	0.52	1.03	1.16	1.48	1.76	1.82
	SEM	0.01	0.01	0.03	0.03	0.01	0.03	0.05
90% ASW	1	0.12	0.57	1.06	1.19	1.51	1.79	1.92
	2	0.13	0.59	1.13	1.26	1.45	1.95	1.72
	3	0.11	0.55	1.03	1.17	1.32	1.84	1.66
	Mean	0.12	0.57	1.07	1.21	1.43	1.86	1.77
	SEM	0.01	0.01	0.03	0.03	0.06	0.05	0.08
80% ASW	1	0.15	0.53	1.04	1.23	1.47	1.79	1.85
	2	0.12	0.62	1.14	1.25	1.56	1.83	1.81
	3	0.12	0.55	1.07	1.28	1.55	1.88	1.84
	Mean	0.13	0.57	1.08	1.25	1.53	1.83	1.83
	SEM	0.01	0.03	0.03	0.01	0.03	0.03	0.01
70% ASW	1	0.11	0.57	0.99	1.19	1.50	1.79	1.95
	2	0.10	0.61	1.14	1.27	1.57	1.85	1.85
	3	0.11	0.60	1.06	1.27	1.53	1.81	1.97
	Mean	0.11	0.59	1.06	1.24	1.53	1.82	1.92
	SEM	0.00	0.01	0.04	0.03	0.02	0.02	0.04
60% ASW	1	0.13	0.59	1.05	1.22	1.69	1.77	2.04
	2	0.13	0.62	1.14	1.31	1.65	1.90	1.97
	3	0.12	0.63	1.11	1.35	1.66	1.93	2.00
	Mean	0.13	0.61	1.10	1.29	1.67	1.87	2.00
	SEM	0.00	0.01	0.03	0.04	0.01	0.05	0.02
50% ASW	1	0.14	0.55	0.90	1.17	1.52	1.82	1.91
	2	0.15	0.54	1.05	1.30	1.65	1.82	2.00
	3	0.12	0.55	0.98	1.30	1.60	1.86	2.01
	Mean	0.14	0.55	0.98	1.26	1.59	1.83	1.97
	SEM	0.01	0.00	0.04	0.04	0.04	0.01	0.03
