# Supplementary material for: Low-salinity medium for large-scale biomass production of the marine purple photosynthetic bacterium Rhodovulum sulfidophilum
Source: PLoS One. 2025 Jun 24;20(6):e0321821. doi: 10.1371/journal.pone.0321821 (PMC12186965; doi:10.1371/journal.pone.0321821)
Supplement: S3 Table — p values from two-way ANOVA (Dunnett’s test) (GraphPad Prism 9) of OD660 comparing 100% with decreasing concentrations of ASW in Fig 1a. (PDF) [file pone.0321821.s003.pdf]

S3 Table.

p	100% ASW						
	0 hours	24 hours	48.5 hours	72 hours	96 hours	128 hours	176.5 hours
90% ASW	>0.9999	0.7153	0.7153	0.7153	0.6602	0.1042	0.6043
80% ASW	>0.9999	0.7683	0.5487	0.1235	0.6602	0.3476	0.9980
70% ASW	0.9874	0.3476	0.8624	0.1992	0.5487	0.6043	0.0730
60% ASW	>0.9999	0.1457	0.3052	0.0115	0.0001	0.0730	0.0002
50% ASW	0.9995	0.9755	0.6602	0.1042	0.0412	0.3476	0.0027
