# Supplementary material for: Low-salinity medium for large-scale biomass production of the marine purple photosynthetic bacterium Rhodovulum sulfidophilum
Source: PLoS One. 2025 Jun 24;20(6):e0321821. doi: 10.1371/journal.pone.0321821 (PMC12186965; doi:10.1371/journal.pone.0321821)
Supplement: S4 Table — Dry cell yield (g L-1) of R. sulfidophilum in ASW supplemented with 0.1% yeast extract and 0.5% peptone in decreasing concentrations of ASW, i.e., 100%, 90%, 80%, 70%, 60%, and 50% which correspond to 3%, 2.7%, 2.4%, 2.1%, 1.8%, and 1.5% salinities respectively (Fig 1b). Data are presented for three independent 15 mL batch cultures (n = 3). P values were obtained from one-way ANOVA (Dunnett’s test) (GraphPad Prism 9) by comparing 100% with decreasing concentrations of ASW. (PDF) [file pone.0321821.s004.pdf]

S4 Table.

Dry cell yield (g L^{-1})					
Treatments	Replicates	Dry cell yield	Mean	SEM	*p*
100% ASW	1	1.50	1.52	0.05	
	2	1.62			
	3	1.45			
90% ASW	1	1.54	1.69	0.10	0.2971
	2	1.88			
	3	1.66			
80% ASW	1	1.73	1.68	0.06	0.3629
	2	1.56			
	3	1.75			
70% ASW	1	1.57	1.70	0.07	0.2679
	2	1.73			
	3	1.80			
60% ASW	1	1.63	1.64	0.01	0.6989
	2	1.65			
	3	1.65			
50% ASW	1	1.80	1.80	0.01	0.0813
	2	1.78			
	3	1.82			
