# Supplementary material for: Low-salinity medium for large-scale biomass production of the marine purple photosynthetic bacterium Rhodovulum sulfidophilum
Source: PLoS One. 2025 Jun 24;20(6):e0321821. doi: 10.1371/journal.pone.0321821 (PMC12186965; doi:10.1371/journal.pone.0321821)
Supplement: S5 Table — Dry cell yield (g L-1) of R. sulfidophilum in ASW supplemented with 1 mM sodium acetate and 2.5 mM sodium thiosulfate in decreasing concentrations of ASW, i.e., 100%, 90%, 80%, 70%, 60%, and 50% which correspond to 3%, 2.7%, 2.4%, 2.1%, 1.8%, and 1.5% salinities respectively (Fig 1c). Data are presented for three independent 15 mL batch cultures (n = 3). P values were obtained from one-way ANOVA (Dunnett’s test) (GraphPad Prism 9) by comparing 100% with decreasing concentrations of ASW. (PDF) [file pone.0321821.s005.pdf]

S5 Table.

Dry cell yield (g L^{-1})					
Treatments	Replicates	Dry cell yield	Mean	SEM	*p*
100% ASW	1	0.18	0.17	0.01	
	2	0.14			
	3	0.18			
90% ASW	1	0.24	0.19	0.03	0.7079
	2	0.20			
	3	0.14			
80% ASW	1	0.16	0.17	0.01	>0.9999
	2	0.19			
	3	0.16			
70% ASW	1	0.14	0.17	0.01	>0.9999
	2	0.19			
	3	0.17			
60% ASW	1	0.20	0.21	0.02	0.2492
	2	0.20			
	3	0.24			
50% ASW	1	0.19	0.22	0.02	0.1614
	2	0.24			
	3	0.23			
