# Supplementary material for: Low-salinity medium for large-scale biomass production of the marine purple photosynthetic bacterium Rhodovulum sulfidophilum
Source: PLoS One. 2025 Jun 24;20(6):e0321821. doi: 10.1371/journal.pone.0321821 (PMC12186965; doi:10.1371/journal.pone.0321821)
Supplement: S6 Table — Dry cell yield (g L-1) of R. sulfidophilum in ASW supplemented with 0.1% yeast extract and 0.5% peptone at 100%, 50%, 40%, and 30% ASW, corresponding to 3%, 1.5%, 1.2%, and 0.9% salinities, respectively. (Fig 2b). Data are presented for four independent 10 L batch cultures (n = 4). P values were obtained from one-way ANOVA (Dunnett’s test) (GraphPad Prism 9) by comparing 100% with decreasing concentrations of ASW. (PDF) [file pone.0321821.s006.pdf]

S6 Table.

Dry cell yield (g L^{-1})					
Treatments	Replicates	Dry cell yield	Mean	SEM	*p*
100% ASW	1	1.07	1.16	0.04	
	2	1.25			
	3	1.21			
	4	1.12			
50% ASW	1	1.24	1.26	0.05	0.5433
	2	1.34			
	3	1.33			
	4	1.14			
40% ASW	1	1.31	1.29	0.03	0.3907
	2	1.25			
	3	1.23			
	4	1.35			
30% ASW	1	1.28	0.99	0.10	0.1592
	2	1.00			
	3	0.83			
	4	0.85			
