# Supplementary material for: Low-salinity medium for large-scale biomass production of the marine purple photosynthetic bacterium Rhodovulum sulfidophilum
Source: PLoS One. 2025 Jun 24;20(6):e0321821. doi: 10.1371/journal.pone.0321821 (PMC12186965; doi:10.1371/journal.pone.0321821)
Supplement: S7 Table — Initial and final OD at 660 nm for 100% and 40% ASW treatments at 10 L scale (Fig 2c). Data are presented for three independent 10 L batch cultures (n = 3). P values were obtained using Student’s T-test statistic (Microsoft Excel 2019) by comparing 100% and 40% ASW treatments at the start of culture and the day of harvest. (PDF) [file pone.0321821.s007.pdf]

S7 Table.

	OD_{660}			
	Day 0		Day 4	
	100% ASW	40% ASW	100% ASW	40% ASW
1	0.080	0.080	1.540	1.990
2	0.080	0.076	1.500	1.650
3	0.072	0.072	1.760	1.880
Mean	0.077	0.076	1.600	1.840
SEM	0.003	0.002	0.081	0.100
p	0.725		0.136	
