# Supplementary material for: Low-salinity medium for large-scale biomass production of the marine purple photosynthetic bacterium Rhodovulum sulfidophilum
Source: PLoS One. 2025 Jun 24;20(6):e0321821. doi: 10.1371/journal.pone.0321821 (PMC12186965; doi:10.1371/journal.pone.0321821)
Supplement: S8 Table — Dry cell yield (g L-1) of R. sulfidophilum in 100% and 40% ASW treatments at 10 L scale (Fig 2d). Data are presented for three independent 10 L batch cultures (n = 3). P values were obtained using Student’s T-test statistic (Microsoft Excel 2019) by comparing 100% and 40% ASW treatments at the start of culture and the day of harvest. (PDF) [file pone.0321821.s008.pdf]

S8 Table.

	Dry cell yield (g L^{-1})			
	Day 0		Day 4	
	100% ASW	40% ASW	100% ASW	40% ASW
1	0.020	0.060	0.850	1.050
2	0.030	0.040	0.800	0.900
3	0.060	0.040	0.950	1.020
Mean	0.037	0.047	0.867	0.990
SEM	0.012	0.007	0.044	0.046
p	0.507		0.124	
