# Supplementary material for: Low-salinity medium for large-scale biomass production of the marine purple photosynthetic bacterium Rhodovulum sulfidophilum
Source: PLoS One. 2025 Jun 24;20(6):e0321821. doi: 10.1371/journal.pone.0321821 (PMC12186965; doi:10.1371/journal.pone.0321821)
Supplement: S9 Table — Growth parameters of R. sulfidophilum in 100% or 40% ASW supplemented with 10 mM sodium thiosulfate pentahydrate and CO2/N2 (7:3) gas mixture (Figs 3b and 3d). Technical replicates are not available for these measurements. (PDF) [file pone.0321821.s009.pdf]

S9 Table.

Growth parameters of *R. sulfidophilum*						
		0 hours	24 hours	47.5 hours	72 hours	95.5 hours
40% ASW	OD_{660}	0.085	0.315	0.710	0.787	0.687
	μ		1.310	0.813	0.103	-0.136
100% ASW	OD_{660}	0.078	0.253	0.610	0.785	0.643
	μ		1.177	0.880	0.252	-0.200
