# Supplementary material for: Low-salinity medium for large-scale biomass production of the marine purple photosynthetic bacterium Rhodovulum sulfidophilum
Source: PLoS One. 2025 Jun 24;20(6):e0321821. doi: 10.1371/journal.pone.0321821 (PMC12186965; doi:10.1371/journal.pone.0321821)
Supplement: S10 Table — Dry cell yield (g L-1) of R. sulfidophilum in 100% or 40% ASW supplemented with 10 mM sodium thiosulfate pentahydrate and CO2/N2 (7:3) gas mixture (Fig 3c). Data are presented for a single 2 L batch culture with 3 technical replicates (n = 3). P values were obtained using Student’s T-test statistic (Microsoft Excel 2019) by comparing 100% and 40% ASW treatments at corresponding time points. n.d. indicates no data. (PDF) [file pone.0321821.s010.pdf]

S10 Table.

Dry cell yield (g L^{-1})						
		0 hours	24 hours	48 hours	72 hours	96 hours
40% ASW	1	0.020	0.133	0.347	0.407	0.367
	2	0.027	0.167	0.327	0.420	0.400
	3	0.060	0.173	0.327	0.433	0.393
	Mean	0.036	0.158	0.333	0.420	0.387
	SEM	0.012	0.012	0.007	0.008	0.010
100% ASW	1	n.d.	0.120	0.273	0.360	0.340
	2	n.d.	0.120	0.247	0.380	0.447
	3	0.033	0.107	0.287	0.400	0.413
	Mean	0.033	0.116	0.269	0.380	0.400
	SEM	0.000	0.004	0.012	0.012	0.032
p		0.187	0.033	0.009	0.045	0.708
