# Supplementary material for: Low-salinity medium for large-scale biomass production of the marine purple photosynthetic bacterium Rhodovulum sulfidophilum
Source: PLoS One. 2025 Jun 24;20(6):e0321821. doi: 10.1371/journal.pone.0321821 (PMC12186965; doi:10.1371/journal.pone.0321821)
Supplement: S11 Table — Total organic carbon (TOC) and total nitrogen (TN) (g L-1) in R. sulfidophilum cells in 100% or 40% ASW supplemented with 10 mM sodium thiosulfate pentahydrate and CO2/N2 (7:3) gas mixture (Figs 3e and 3f). Data are presented for a single 2 L batch culture with 3 technical replicates (n = 3). P values were obtained using Student’s T-test statistic (Microsoft Excel 2019) by comparing 100% and 40% ASW treatments at corresponding time points. (PDF) [file pone.0321821.s011.pdf]

S11 Table.

Cell TOC and TN (mg L^{-1})							
			0 hours	24 hours	48 hours	72 hours	96 hours
40% ASW	Cell TOC	1	33.5	87.4	178.1	203.0	195.5
		2	35.1	84.6	186.5	211.2	201.7
		3	33.7	79.4	185.2	211.0	198.8
		Mean	34.1	83.8	180.8	208.4	198.7
		SEM	0.5	2.3	2.6	2.7	1.8
	Cell TN	1	6.7	17.3	34.4	41.5	41.6
		2	6.5	17.8	35.2	41.6	43.0
		3	6.4	17.9	35.0	41.3	42.5
		Mean	6.6	17.7	34.9	41.5	42.4
		SEM	0.1	0.2	0.2	0.1	0.4
100% ASW	Cell TOC	1	34.3	70.3	150.6	204.8	209.2
		2	32.0	66.7	157.1	204.2	208.3
		3	29.1	66.3	155.3	203.8	206.4
		Mean	31.8	67.7	154.3	204.3	208.0
		SEM	1.5	1.3	1.9	0.3	0.8
	Cell TN	1	6.2	11.3	24.7	35.8	38.1
		2	6.5	10.7	24.6	35.9	39.2
		3	5.7	11.4	24.2	35.9	39.4
		Mean	6.2	11.1	24.5	35.8	38.5
		SEM	0.2	0.2	0.2	0.0	0.4
p	Cell TOC		0.221	0.004	0.001	0.204	0.009
	Cell TN		0.181	2.4E-05	3.2E-06	5.4E-07	0.004
