# Supplementary material for: Low-salinity medium for large-scale biomass production of the marine purple photosynthetic bacterium Rhodovulum sulfidophilum
Source: PLoS One. 2025 Jun 24;20(6):e0321821. doi: 10.1371/journal.pone.0321821 (PMC12186965; doi:10.1371/journal.pone.0321821)
Supplement: Attachment — Submitted filename: Response to reviewers_clean.doc [file pone.0321821.s013.doc]

Reviewers' comments:

Reviewer #1: The authors of this study explore a cost-effective alternative to nutrient-rich culture media for large-scale biomass production of the marine purple photosynthetic bacterium Rhodovulum sulfidophilum, which is valuable in bioremediation, biotechnology, and agriculture. The researchers found that reducing salinity in seawater-based cultures from 3% to 1.2% did not negatively impact heterotrophic growth, cell yield, nitrogen content, or amino acid composition. Lower salinity also proved feasible for cultivation without raising contamination risks, offering a viable option for large-scale use as a plant nitrogen fertilizer. However, there are some concerns, as listed below:

Response: We thank you for the careful review of our manuscript and appreciate your efforts in improving it.

a) The authors should study the long-term stability and viability of R. sulfidophilum cultures at reduced salinity levels.

Response: We acknowledge that the current study primarily addresses short-term growth response under reduced salinity conditions. However, similar growth (Figs 2c and 3b) and dry cell yield (Figs 2d and 3c) in low salinities (1.2%) demonstrate that the cells are metabolically active and viable to the same degree as 3% salinity, which supports the effectiveness of the modified medium. Moreover, we did not observe any visible signs of reduced viability or stress, such as loss of pigments or culture collapse, during the experimental timeline. Thus, there are no indicators that point towards a possible negative impact on the long-term stability and viability of this bacterium. However, we agree that long-term subculturing and monitoring is an important direction of future work and have mentioned this in the discussion (Page 11, lines 163 to 168).

Page 11, lines 163 to 168

Thus, the culture medium for large-scale cultivation of *R. sulfidophilum* was comprehensively improved without any trade-offs. However, this study primarily focused on short-term growth responses under reduced salinity conditions. To assess the feasibility of this approach for sustained industrial use, further evaluation of long-term culture stability and viability across multiple subculturing cycles may be necessary.

b) The authors should comment on the environmental impact of using low-salinity seawater for large-scale cultivation.

Response: We have briefly discussed the environmental impact of low-salinity seawater medium for large-scale cultivation (Page 12, lines 200 to 207)

Page 12, lines 200 to 207

Finally, the use of low-salinity ASW-based growth medium offers environmental advantages over conventional nutrient-rich formulations. These include reduced nutrient input and discharge, thereby lowering the risk of eutrophication in vulnerable aquatic systems, as well as decreasing salinity in waste streams, which simplifies downstream treatment and disposal. Leveraging the autotrophic growth of *R. sulfidophilum* could further reduce dependency on organic inputs, lowering the demand on land, water, and animal-derived byproducts, thus decreasing the carbon and energy footprint associated with biomass production.

c) The authors should provide a zoom in picture of Fig1A to clearly distinguish the difference of OD600 at 175 hours of culture

Response: A zoomed inlay of Fig 1a at 176.5 hours is now included for better data visualization.

**Fig 1.** Heterotrophic cell growth and dry cell yield of *Rhodovulum sulfidophilum* in artificial seawater (ASW) based growth medium of different salinities, wherein 100% corresponds to 3% salinity.

**a,** Growth curve and **b,** dry cell yield (g L^-1^) of *R. sulfidophilum* in ASW supplemented with 0.1% yeast extract and 0.5% peptone, and **c,** dry cell yield (g L^-1^) of *R. sulfidophilum* in ASW supplemented with 1 mM sodium acetate and 2.5 mM sodium thiosulfate in decreasing concentrations of ASW i.e., 100%, 90%, 80%, 70%, 60%, and 50% which correspond to 3%, 2.7%, 2.4%, 2.1%, 1.8%, and 1.5% salinities respectively. Zoomed inlay in Fig 1a shows OD_660_ for each treatment at 176.5 hours. Data represent means ± SEM for 3 independent 15 mL batch cultures (n=3). Asterisks indicate significant differences between 100% and decreasing concentrations of ASW tested using one-way ANOVA (Figs 1b and 1c) or two-way ANOVA (Fig 1a), and the Dunnett’s multiple comparison test (GraphPad Prism 9); * p<0.05, ** p<0.01, and *** p<0.001.

d) The authors should confirm the number of trials, especially for 60 and 50% ASW, in Fig 1b

Response: The values shown in Figs 1a, b and c represent averages from three independent cultures and are originally mentioned (highlighted in blue) in the figure legend for Fig 1 as shown above.

e) Figs. 2 and 3 need clarification on statistics, and the authors should provide all the raw data and statistics. Fig 2c,d need statistics and Fig 3e requires further explanation of which culture performed better.

Response: The raw data and statistics used for the graphs in the figures and tables are now provided in supporting information files and the table and figure legends. Further explanation of results from Fig 3e are now included (Pages 9 and 10 , lines 135 to 140)

Table and figure legends

**Table 1.** Cell yield and cost analysis of various growth media for large-scale cultivation of *Rhodovulum sulfidophilum*.

Data represent means ± SEM from 4 independent 10 L cultures, or batches (n=4). Different letters indicate the significance of differences observed with Marine Broth (MB) as evaluated by a one-way ANOVA (Dunnett’s test) (GraphPad Prism 9).

**Table 2.** Free and total amino acid profile expressed in weight % of lysed and dried biomass (Processed Biomass or PB) from *R. sulfidophilum* cultured in 100% and 40% ASW.

Data are shown for the combined PB from 3 independent batch cultures; error bars not shown as statistical variation could not be calculated from pooled data. Values should be interpreted as representative trends. n.d. indicates no data due to degradation by HCl

**Fig 1.** Heterotrophic cell growth and dry cell yield of *Rhodovulum sulfidophilum* in artificial seawater (ASW) based growth medium of different salinities, wherein 100% corresponds to 3% salinity.

**a,** Growth curve and **b,** dry cell yield (g L^-1^) of *R. sulfidophilum* in ASW supplemented with 0.1% yeast extract and 0.5% peptone, and **c,** dry cell yield (g L^-1^) of *R. sulfidophilum* in ASW supplemented with 1 mM sodium acetate and 2.5 mM sodium thiosulfate in decreasing concentrations of ASW i.e., 100%, 90%, 80%, 70%, 60%, and 50% which correspond to 3%, 2.7%, 2.4%, 2.1%, 1.8%, and 1.5% salinities respectively. Zoomed inlay in Fig 1a shows OD_660_ for each treatment at 176.5 hours. Data represent means ± SEM for 3 independent 15 mL batch cultures (n=3). Asterisks indicate significant differences between 100% and decreasing concentrations of ASW tested using one-way ANOVA (Figs 1b and 1c) or two-way ANOVA (Fig 1a), and the Dunnett’s multiple comparison test (GraphPad Prism 9); * p<0.05, ** p<0.01, and *** p<0.001.

**Fig 2.** Dry cell yields and contamination status in ASW-based *R. sulfidophilum* cultures at a reduced salinity of 1.2%; 100% and 40% correspond to 3% and 1.2% salinity respectively.

**a,** Ten-liter culture set-up and **b,** dry cell yield (g L^-1^) of *R. sulfidophilum* in ASW supplemented with 0.1% yeast extract and 0.5% peptone at 100%, 50%, 40%, and 30% ASW, corresponding to 3%, 1.5%, 1.2%, and 0.9% salinities respectively. **c,** Initial and final OD at 660 nm and **d,** the dry cell yield (g L^-1^) were obtained for 100% and 40% ASW treatments. **e,** Contamination status at the end of the culture period was obtained for 10^-6^ to 10^-8^ serial dilutions of 100% (*left panel*) and 40% (*right panel*) ASW treatments. Data represent means ± SEM for n=3 or 4. Significant differences between the 3% and 1.2% salinity treatments were not observed using a one-way ANOVA (Dunnett’s test) (GraphPad Prism 9). Red circles indicate the contaminating colonies.

**Fig 3.** Cell growth, dry cell yield, total organic carbon (TOC), and total nitrogen (TN) in autotrophic cultures of *R. sulfidophilum* in artificial seawater (ASW) based growth medium of different salinities, wherein 100% corresponds to 3% salinity and 40% corresponds to 1.2% salinity.

**a,** Two-liter fermenter set-up for the culture of *R. sulfidophilum* in 100% or 40% ASW supplemented with 10 mM sodium thiosulfate pentahydrate and CO_2_/N_2_ (7:3) gas mixture. **b,** Optical density (OD) at 660 nm, **c,** dry cell yield (g L^-1^), and **d,** OD-based specific growth rates (*µ*) were obtained for every day of the culture. The total C and N in the cell fractions are shown as **e,** cell TOC (mg L^-1^), and **f,** cell TN (mg L^-1^) respectively. Data represent means ± SEM from three technical replicates (n=3). Asterisks indicate significant differences between the 3% and 1.2% salinity treatments using the Student’s T-test statistic (Microsoft Excel 2019); * p<0.05, ** p<0.01, and *** p<0.001.

Pages 9 and 10 , lines 135 to 140

TOC of cells in 1.2% salinity was higher during initial growth (Fig 3e and Table S11), consistent with cell growth (Figs 3b and 3c). However, the TN remained higher in cells throughout the culture in 1.2% salinity compared to 3% salinity (Fig 3f and Table S11). Thus, 1.2% salinity was more effective than 3% salinity in supporting autotrophic growth of *R. sulfidophilum* in ASW-based growth medium.

Reviewer #2:

Response: We thank you for the careful review of our manuscript and appreciate your efforts in improving it.

Strengths:
• Clearly defined experimental setup across heterotrophic and autotrophic conditions.
• Comprehensive detail on salinity conditions, growth curve, biomass harvesting, and chemical analysis.
• Inclusion of contamination check and sterilization methods shows good microbiological practice.
• The use of seawater and cost-effective flocculation steps shows practical relevance.

Some lacks:
1. Does the study present actual plant growth trails under greenhouse or field conditions using the biomass obtained from 1.2%?

Response: In our previous study, we demonstrated the use of biomass from 3% salinity as an effective nitrogen (N) fertilizer for cultivation of Japanese mustard spinach in greenhouse conditions. The N content (% w/w) of biomass obtained from 1.2% salinity is similar to the N content of the biomass obtained from 3% salinity, thus indicative of lack of effects on total N and amino acid content due to reduced salinity. For this reason, we do not show plant growth trials under greenhouse or field conditions in this study. We have included this in the discussion (Page 12, lines 185 to 189)

Page 12, lines 185 to 189

Previously, we have shown that PB from MB (N : P : K = 11.0 : 2.95 : 0.51) can be effectively used as N fertilizer for the cultivation of Japanese mustard spinach (komatsuna) (18). In this study, PB obtained from 1.2% salinity medium showed comparable N : P : K of 11.9 : 2.94 : 0.57, indicating its feasibility for use as a plant N fertilizer.

2. The author mentioned “cost effective”. So, the author should provide details.

Response: The cost-effectiveness of 1.2% salinity medium is now included in the text (Page 7, lines 100 to 102).

Page 7, lines 100 to 102

Additionally, decreasing the salinity of the ASW-based culture medium by 60% (3% to 1.2%) decreased culture costs to 92% and 12% relative to MB and NSW-based culture medium, respectively.

3. Does the study assess the long-term environmental impact or biodegradability of the biomass when used as fertilizer?

Response: The current goal of this study is only an evaluation of 1.2% salinity for biomass production. Thus, we haven’t evaluated the long-term impact or biodegradability of the biomass when used as a fertilizer. We agree with reviewer #2 that such an assessment is imperative for its sustained use as fertilizer, and we plan to make these evaluations in the future. However, we believe this is beyond the primary goals of the current study.

4. Lysed biomass as fertilizer (lines 14–15) and again in lines 16–18 feels slightly repetitive.

Response: This section has been revised accordingly (Page 3, lines 16 to 23)

Page 3, lines 16 to 23

Additionally, we have reported that its lysed and dried biomass can be used as a sustainable N fertilizer for plant cultivation due to its N content of ~11% and protein content of ~69% (18). This allows biomass waste to be repurposed as plant fertilizer after the extraction of value-added compounds.

5. The "gap" could be emphasized more for a strong impact. If it is possible.

Response: We are unsure what “gap” the reviewer is referring to here and hence are unable to address this comment.

6. Citations (e.g., 1–18) are referenced extensively, but some seem clustered without specifics. It's unclear which statements each citation supports.

Response: We believe we have been specific with the references, following the parts of sentences with exact references instead of including references at the end of each sentence. We would appreciate it if you could please be more specific about the clustered section in question here.

7. Line 161: “R. sulfidophilum (DSM 1374; W4, LMG 5202) (ATCC)” is slightly confusing.
8. Suggestion: Clarify the exact strain and source used:
“Rhodovulumsulfidophilum (DSM 1374, equivalent to LMG 5202; obtained from ATCC) was used for all cultivation experiments.”
If multiple strains or IDs are applicable, clarify which is the principal one.

Response for comments 7 and 8: We have changed the sentence as suggested (Page 13, lines 209 to 212)

Page 13, lines 209 to 212

**Cultivation of *Rhodovulum sulfidophilum***

*Rhodovulum sulfidophilum* (DSM 1374; equivalent to LMG 5202 obtained from ATCC) was used for all experiments in this study, and cultivated in various media with different salt compositions as detailed below.

Reviewer #3: Peer Review Report

Response: We thank you for the careful consideration of our manuscript and appreciate your efforts in improving it.

Recommendation: Minor Revision
This manuscript presents a well-structured and carefully executed study that investigates the potential for using a low-salinity artificial seawater medium for the large-scale cultivation of Rhodovulumsulfidophilum. The work is highly relevant to sustainable biotechnology, particularly for cost-effective biomass production with applications in biofertilizers.

Q1. Technical Soundness and Support for Conclusions
Yes. The experiments were thoughtfully designed and appropriately replicated (e.g., four biological replicates for 10 L batch cultures), with robust comparisons across conventional marine broth and both natural and artificial seawater-based media. The core findings—that a salinity reduction from 3% to 1.2% does not compromise growth, biomass yield, or biomass quality—are well supported by both qualitative observations and quantitative data. The authors also checked for contamination and demonstrated that low salinity did not increase contamination risk. Overall, the conclusions are well grounded in the evidence provided.

Q2. Statistical Analysis
Yes. The manuscript demonstrates sound use of statistics. Two-way ANOVA with post-hoc Tukey tests is used appropriately for comparisons across salinities and media types, while Student’s t-tests are applied to pairwise comparisons. The authors provide mean values and SEM, and statistical significance is clearly indicated throughout the figures. One suggestion would be to clarify in the text whether phrases like “tended to be higher” refer to statistically significant differences or trends that did not reach significance.

Response: We have considered the suggestion and made the changes accordingly (see response to comments #1 and #3 below)

Q3. Data Availability
Yes. The authors clearly state that all data underlying the results are included in the manuscript and supporting information files. Key results are well-documented in figures and tables, with no apparent omissions. The study complies with PLOS ONE’s data availability policies.

Q4. Clarity of Presentation
Yes. The manuscript is generally well-written, with clear and logical flow. The introduction provides adequate context and rationale, and the results and discussion are well organized. While the English is fluent and professional, a light proofreading for minor typographical inconsistencies would be beneficial. For example, compound words like “cost-effective” and unit formatting (e.g., “g L⁻¹” vs “g L-1”) should be made consistent throughout the manuscript.

Response: Thank you for the suggestion. We have formatted the manuscript as requested and embedded fonts to prevent changes between file formats. (see response to comment #5 below)

Review Comments to the Authors

General Assessment:
This study is a valuable contribution to microbial biotechnology and sustainable agriculture. It offers a practical solution to reduce media cost in large-scale microbial cultivation without sacrificing performance or introducing new risks. The use of both heterotrophic and autotrophic growth conditions strengthens the applicability of the findings.
Strengths:
• The stepwise experimental design—starting with media comparison and moving toward salinity optimization—is a strong feature.
• The cost analysis is timely and relevant for scaling microbial production.
• The consistent performance of the 1.2% salinity medium under both heterotrophic and autotrophic conditions is clearly demonstrated.
• Contamination checks, amino acid profiling, and N:P:K analysis add meaningful depth to the study.
•
Suggestions for Improvement:
1. Statistical clarity: Please indicate in the text whether observed differences (e.g., higher N content or amino acid levels at 1.2% salinity) are statistically significant or simply trends. If tests were not applied to pooled amino acid data, a note to that effect would be helpful.

Response: The text has been modified for statistical clarity (Page 7, lines 92 to 100). The legend of Table 2 has been modified accordingly (see response to comment #3).

Page 7, lines 92 to 100

The N:P:K of PB from 40% ASW treatment was 11.9 : 2.94 : 0.57, comparable to previously reported values in MB (11.0 : 2.95 : 0.51) (18), with a slight tendency toward higher N in the former. The total amino acid composition between the PB from 100% and 40% ASW was comparable, except for glutamate and glutamine, which tended to be higher in 40% ASW (Table 2). The composition of free amino acids, on the other hand, was marginally different, with a trend towards higher lysine, glutamate, and aspartate; and lower arginine in PB from 40% ASW (Table 2).

2. Table 1 presentation: Clarify how “relative dry cell yield” was calculated and consider revising the phrasing around the cost difference to make it more intuitive.

Response: To avoid any confusion, we have deleted the column for relative dry cell yield from Table 1, because significant differences in dry cell yield between MB and seawater treatments were not observed. We have revised the phrasing around the cost difference to make it more intuitive (Page 4, lines 38 to 43)

Page 4, lines 38 to 43

Using NSW- and ASW-based culture media instead of MB decreased the culture media costs by 90% and 91%, respectively (Table 1). Additionally, culturing in the ASW-based medium was 9% cheaper than using the NSW-based culture medium (Table 1).

3. Free amino acid profiles: Since Table 2 presents data from pooled samples, please indicate that error bars are not available and explain briefly how this affects interpretation.

Response: We acknowledge that Table 2 presents data from pooled samples, and hence, error bars cannot be calculated. This limitation and an explanation on data interpretation have now been clarified in the manuscript (Table 2 legend)

**Table 2.** Free and total amino acid profile expressed in weight % of lysed and dried biomass (Processed Biomass or PB) from *R. sulfidophilum* cultured in 100% and 40% ASW.

Data are shown for the combined PB from 3 independent batch cultures; error bars not shown as statistical variation could not be calculated from pooled data. Values should be interpreted as representative trends. n.d. indicates no data due to degradation by HCl.

4. Terminology: In the Abstract or Introduction, consider stating that 3% NaCl is equivalent to the salinity of natural seawater. Readers from outside marine microbiology may not know this.

Response: We have included this in the introduction as suggested (Page 3, lines 8 to 11)

Page 3, lines 8 to 11

These bacteria are reported to tolerate a wide range of salinity (4,11,12) and are predominantly grown in culture medium with 2% – 3% NaCl (7,9,10,12–15). The use of natural seawater (approximately 3% – 3.5% salinity) for their culture has been previously proposed for the sustainable, cost-effective production of these value-added compounds with a low risk of biological contamination (13,16,17).

5. Formatting and style: Ensure consistent formatting for units and superscripts in figure legends (e.g., “10⁻⁶” rather than “10-6”).

Response: We have confirmed that the formatting is consistent for units in figure legends and embedded fonts to ensure compatibility across different file formats. (Figure legends shown in response to comment #6)

6. Figure legends: Please make sure each figure legend explains the basis of statistical comparisons—e.g., whether comparisons are across all salinities or between two specific groups.

Response: We have revised the table and figure legends to clarify this.

**Table 1.** Cell yield and cost analysis of various growth media for large-scale cultivation of *Rhodovulum sulfidophilum*.

Data represent means ± SEM from 4 independent 10 L cultures, or batches (n=4). Different letters indicate the significance of differences observed with Marine Broth (MB) as evaluated by a one-way ANOVA (Dunnett’s test) (GraphPad Prism 9).

**Table 2.** Free and total amino acid profile expressed in weight % of lysed and dried biomass (Processed Biomass or PB) from *R. sulfidophilum* cultured in 100% and 40% ASW.

Data are shown for the combined PB from 3 independent batch cultures; error bars not shown as statistical variation could not be calculated from pooled data. Values should be interpreted as representative trends. n.d. indicates no data due to degradation by HCl

**Fig 1.** Heterotrophic cell growth and dry cell yield of *Rhodovulum sulfidophilum* in artificial seawater (ASW) based growth medium of different salinities, wherein 100% corresponds to 3% salinity.

**a,** Growth curve and **b,** dry cell yield (g L^-1^) of *R. sulfidophilum* in ASW supplemented with 0.1% yeast extract and 0.5% peptone, and **c,** dry cell yield (g L^-1^) of *R. sulfidophilum* in ASW supplemented with 1 mM sodium acetate and 2.5 mM sodium thiosulfate in decreasing concentrations of ASW i.e., 100%, 90%, 80%, 70%, 60%, and 50% which correspond to 3%, 2.7%, 2.4%, 2.1%, 1.8%, and 1.5% salinities respectively. Zoomed inlay in Fig 1a shows OD_660_ for each treatment at 176.5 hours. Data represent means ± SEM for 3 independent 15 mL batch cultures (n=3). Asterisks indicate significant differences between 100% and decreasing concentrations of ASW tested using one-way ANOVA (Figs 1b and 1c) or two-way ANOVA (Fig 1a), and the Dunnett’s multiple comparison test (GraphPad Prism 9); * p<0.05, ** p<0.01, and *** p<0.001.

**Fig 2.** Dry cell yields and contamination status in ASW-based *R. sulfidophilum* cultures at a reduced salinity of 1.2%; 100% and 40% correspond to 3% and 1.2% salinity respectively.

**a,** Ten-liter culture set-up and **b,** dry cell yield (g L^-1^) of *R. sulfidophilum* in ASW supplemented with 0.1% yeast extract and 0.5% peptone at 100%, 50%, 40%, and 30% ASW, corresponding to 3%, 1.5%, 1.2%, and 0.9% salinities respectively. **c,** Initial and final OD at 660 nm and **d,** the dry cell yield (g L^-1^) were obtained for 100% and 40% ASW treatments. **e,** Contamination status at the end of the culture period was obtained for 10^-6^ to 10^-8^ serial dilutions of 100% (*left panel*) and 40% (*right panel*) ASW treatments. Data represent means ± SEM for n=3 or 4. Significant differences between the 3% and 1.2% salinity treatments were not observed using a one-way ANOVA (Dunnett’s test) (GraphPad Prism 9). Red circles indicate the contaminating colonies.

**Fig 3.** Cell growth, dry cell yield, total organic carbon (TOC), and total nitrogen (TN) in autotrophic cultures of *R. sulfidophilum* in artificial seawater (ASW) based growth medium of different salinities, wherein 100% corresponds to 3% salinity and 40% corresponds to 1.2% salinity.

**a,** Two-liter fermenter set-up for the culture of *R. sulfidophilum* in 100% or 40% ASW supplemented with 10 mM sodium thiosulfate pentahydrate and CO_2_/N_2_ (7:3) gas mixture. **b,** Optical density (OD) at 660 nm, **c,** dry cell yield (g L^-1^), and **d,** OD-based specific growth rates (*µ*) were obtained for every day of the culture. The total C and N in the cell fractions are shown as **e,** cell TOC (mg L^-1^), and **f,** cell TN (mg L^-1^) respectively. Data represent means ± SEM from three technical replicates (n=3). Asterisks indicate significant differences between the 3% and 1.2% salinity treatments using the Student’s T-test statistic (Microsoft Excel 2019); * p<0.05, ** p<0.01, and *** p<0.001.

7. Literature integration: References [13] and [18] are cited several times. Consider acknowledging their foundational role in the discussion, especially in relation to media design and previous applications of R. sulfidophilum biomass as fertilizer.

Response: We acknowledge the foundational role of [18] in this study and have now included this in the discussion (Page 12, lines 185 to 189). However, [13] has only been cited twice in the text in relation to culture medium optimization for PHA production in this bacterium under heterotrophic conditions, and we believe this doesn’t play any foundational role for the culture of this bacterium for its use as plant fertilizer. However, we have previously discussed the relationship between PHA accumulation, growth, and salinity, referring to other studies in Page 11, lines 169 to 184 (highlighted in blue).

Page 12, lines 185 to 189

Previously, we have shown that PB from MB (N : P : K = 11.0 : 2.95 : 0.51) can be effectively used as N fertilizer for the cultivation of Japanese mustard spinach (komatsuna) (18). In this study, PB obtained from 1.2% salinity medium displayed comparable N : P : K of 11.9 : 2.94 : 0.57, indicating its feasibility for use as a plant N fertilizer.

Page 11, lines 169 to 184

The trend of higher growth (Fig 1a) and dry cell yield in culture medium with lower salinities (Figs 1b and 1c) is consistent with a previous study, wherein decreased PHA accumulation in cells was also observed with a decrease in salinity from 4.5% to 1.5% (19). Acetyl-CoA is the precursor for PHA biosynthesis in *R. sulfidophilum* (9,15) and also a common metabolic intermediate of the C metabolism feeding into the TCA cycle, which provides C skeletons necessary for N metabolism, including the biosynthesis of amino acids (20). Hence, a decrease in C flux towards PHA biosynthesis could increase the flux towards the TCA cycle, facilitating improved growth and/or N assimilation in the lower salinity growth medium. This was also observed in the current study wherein cells when grown autotrophically, tended to assimilate more N in the 1.2% salinity growth medium (Fig 3f). A similar tendency may also be expected in heterotrophic cultivation leading to marginally higher N content (11.9%) in PB at 1.2% salinity. However, due to the difference in the modes of metabolism, further study would be necessary to verify the mechanism of a higher N utilization and N content in a 1.2% salinity medium under respective growth conditions.
